# Volatile Organic Compounds of Bryophytes from Peninsular Malaysia and Their Roles in Bryophytes

**DOI:** 10.3390/plants11192575

**Published:** 2022-09-29

**Authors:** Chin Wen Koid, Nur Fariza M. Shaipulah, Gaik Ee Lee, S. Robbert Gradstein, Yoshinori Asakawa, Yosie Andriani, Arifullah Mohammed, Nik Norhazrina, Poh Wai Chia, Muhammad Zulhimi Ramlee

**Affiliations:** 1 Faculty of Science and Marine Environment, Universiti Malaysia Terengganu, Kuala Nerus 21030, Terengganu, Malaysia; 2 Institute of Tropical Biodiversity and Sustainable Development, Universiti Malaysia Terengganu, Kuala Nerus 21030, Terengganu, Malaysia; 3 Meise Botanic Garden, 1860 Meise, Belgium; 4 Institute of Pharmacognosy, Tokushima Bunri University, Tokushima 770-8514, Japan; 5 Institute of Marine Biotechnology, Universiti Malaysia Terengganu, Kuala Nerus 21030, Terengganu, Malaysia; 6 Department of Agriculture Science, Faculty of Agro-Based Industry, Universiti Malaysia Kelantan, Jeli 17600, Kelantan, Malaysia; 7 Department of Biological Sciences and Biotechnology, Faculty of Science and Technology, Universiti Kebangsaan Malaysia, Bangi 43600, Selangor, Malaysia; 8 Centre of Research and Field Service (CRaFS), Universiti Malaysia Terengganu, Kuala Nerus 21030, Terengganu, Malaysia

**Keywords:** mosses, liverworts, hornworts, elevational differentiation of bryophytes, chemical evolution, volatile organic compounds, GC-MS, Peninsular Malaysia

## Abstract

Volatile emissions from 22 bryophyte species from Peninsular Malaysia were collected using a dynamic headspace technique and analyzed by gas chromatography–mass spectrometry (GC-MS). Thirty organic compounds (VOCs) from eight different groups were detected in bryophytes from the montane forest in Cameron Highlands and the lowland dipterocarp forest in Lata Belatan. The headspace of bryophytes in Cameron Highlands was dominated by tetradecane, 2-ethyl-1-hexanol, decanal, pentanoic acid, 2,2,4-trimethyl-3-carboxyisopropyl, isobutyl ester, D-limonene and naphthalene. On the contrary, in the bryophyte headspace of Lata Belatan, naphthalene and tetradecane were dominant compounds. The elevational pattern detected in VOC composition of bryophytes appears to be an evolutionary feature at the family level that needs verification at other sites. The results also confirmed that the VOC composition of bryophytes is species-specific. The roles of VOCs in bryophytes are presented, including plant–plant communication and plant–insect interaction and as an additional taxonomic character in chemotaxonomy.

## 1. Introduction

Volatile organic compounds (VOCs) are one of plants′ most important secondary metabolites. They are metabolic products or byproducts with high vapor pressure at room temperature and low molecular weight. VOCs are ubiquitous in air, water and soil and are important in mediating intra- and interspecific interactions among organisms in the ecosystem. Although it has long been known that their emission primarily responds to biotic and abiotic stresses involving various ecological functions, including defense, communication, protection and adaptation, many VOCs still have unknown roles. Several studies have highlighted that apart from the ecosystem functional explanation, the physicochemical characteristics of VOCs also play a role in determining the diversity, pattern and quantity of their emissions [1]. VOCs are highly diverse and can be classified according to their biosynthetic origin and chemical structure, including alkanes, alkenes, alcohols, aldehydes, aromatic hydrocarbons, ethers and carboxylic acids. Abundant literature on vascular plant VOCs is available, in particular their contribution to floral scent and mimicry, which play vital roles in plant–insect interaction, e.g., attracting pollinators and imitating mating signals [2,3]. Furthermore, the potential of VOCs in agriculture and as biological control agents in fighting disease is also promising [4]. Although the composition and function of plant VOCs have been investigated for many decades, little is known about the VOCs′ emission and roles in nonvascular plant groups, including green algae and bryophytes. Bryophytes are the second-largest land plants after angiosperms, and they are collectively divided into three main groups: Bryophyta (mosses), Marchantiophyta (liverworts) and Anthocerotophyta (hornworts). Among the bryophytes, liverworts have the most diverse bioactive compounds, of which terpenoids are the most abundant ones, with more than 1600 compounds hitherto identified [5]. The presence of oil bodies, a unique organelle only found in liverworts, has been proposed as a means for storing terpenoids in liverworts [6]. This paper aims to provide VOC profiling of selected Peninsular Malaysian bryophytes and review recent literature on volatile communication between bryophytes and other organisms.

## 2. Results

The volatile compounds were identified using the dynamic headspace and analyzed using gas chromatography–mass spectrometry (GC-MS). A total of 30 chemical compounds were detected from the montane forest in Cameron Highlands and the lowland dipterocarp forest in Lata Belatan. Eleven compounds were detected from both sampling sites and classified into different classes of volatiles: alkane, alkene, aldehyde and aromatic hydrocarbon.

### 2.1. Identification of the Volatile Constituents of Liverworts, Mosses and Hornwort in Cameron Highlands, a Montane Rainforest at 1400–1600 m Elevation

In the headspace of 12 bryophytes species from Cameron Highlands (Table 1), 30 compounds were detected, representing 93.5% to 99.9% of the total emission. Most of the species were mainly composed of alkane and terpene volatiles. The liverwort *Bazzania loricata* emitted the highest number of compounds with 20 volatiles and predominantly contained pentanoic acid, 2,2,4-trimethyl-3-carboxyisopropyl, isobutyl ester, which accounted for 27.5% of the total volatiles, respectively. On the other hand, the moss *Dicranoloma braunii* emitted only seven compounds, the lowest number of volatile components detected per species.

**Figure 1:**
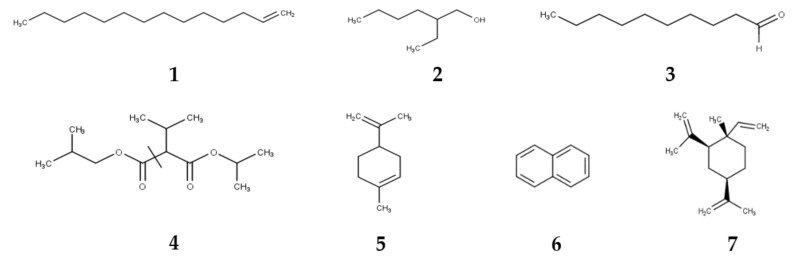
Major volatile compounds found in bryophytes. Abbreviations: 1-tetradecene (**1**), 2-ethyl-1-hexanol (**2**), decanal (**3**), pentanoic acid, 2,2,4-trimethyl-3-carboxyisopropyl, isobutyl ester (**4**), limonene (**5**), naphthalene (**6**), β-elemene (**7**).

The GC-MS analysis of chemical constituents in the headspace of bryophyte samples in Cameron Highlands showed similar chemical composition (Table 2). Six compounds, namely 1-tetradecene (**1**), 2-ethyl-1-hexanol (**2**), decanal (**3**), pentanoic acid, 2,2,4-trimethyl-3-carboxyisopropyl, isobutyl ester (**4**), limonene (**5**) and naphthalene (**6**) were common to all species (Figure 1). Pentanoic acid, 2,2,4-trimethyl-3-carboxyisopropyl, isobutyl ester was the most abundant compound. It occurred in the mosses (*Fissidens crispulus, Distichophyllum mittenii,* *Garovaglia elegans*) and liverworts (*Bazzania loricata, Frullania apiculata*, *Heteroscyphus coalitus*, *Mastigophora diclados, Plicanthus hirtellus*) and hornwort (*Anthoceros angustus*), accounting from 16.9% to 34.6% of total volatiles. 2-ethyl-1-hexanol was identified as the main volatile component in *B. longicaulis* and *Dicranoloma braunii*, representing 26% and 41.2% of total peak areas, respectively. β-elemene (**7**) was recorded as a major component in *Plagiochila bantamensis,* accounting for 17.7% of total volatiles. Naphthalene was present in 11 of 13 species, accounting for 4.1% to 16.6% of total volatiles. Of the other aromatic hydrocarbons, benzothiazole was detected in *H. coalitus* (6.3%), *Frullania apiculata* (3.8%), *Plicanthus hirtellus* (4.1%) and *Dicranoloma braunii* (6.7%), and anethole was exclusively identified in *B. loricata* (2.5%). The monoterpene hydrocarbons, limonene and oxygenated monoterpenes, linalool and menthol were identified in 11 species. Relative areas of limonene were recorded in 11 species that showed a minimum value of 3.3% and a maximum value of 17.4%. Linalool was detected in *H. coalitus* (6.5%), *Frullania apiculata* (5.6%), *B. loricata* (2.7%) and *M. diclados* (4.4%). Menthol was present in *Distichophyllum mittenii* (8.8%), *H. coalitus* (6.9%), *Frullania apiculata* (5.4%) and *Dicranoloma braunii* (4.8%). The headspace of *Plagiochila bantamensis* contained three sesquiterpene volatiles, β-elemene (17.4%), 1,8-dimethyl-4-(prop-1-en—2-yl)spiro [4.5]dec-7-ene (6.5%) and germacrene B (10.4%). Tetradecamethyl- cycloheptasiloxane was detected in *Fissidens crispulus* (15.7%) and *M. diclados* (2.6%). Diterpene volatile, kaur-16-ene was only identified in *B. loricata*. Methyl salicylate, a plant stress compound was present in a small amount in *Fissidens crispulus* (3.2%), *Frullania apiculata* (3.1%), *Plicanthus hirtellus* (2.3%), *B. loricata* (2.6%) and *M. diclados* (2.3%).

### 2.2. Identification of the Volatile Constituents of Liverworts and Moss in Lata Belatan, a Lowland Dipterocarp Forest at 100–200 m Elevation

In nine bryophyte species of Lata Belatan (Table 3), 3 to 11 compounds were identified, accounting for 93.1% to 99.9% of total volatiles (Table 4). Naphthalene (**6**) was found to be the most abundant volatile component in the liverworts *Pycnolejeunea cavistipula* (68.9%), *Drepanolejeunea ternatensis* (67.9%), *Pallavicinia lyellii* (62%) and the moss *Oedicladium pseudorufescens* (67.9%). Alkane dominated *Pycnolejeunea grandiocellata*, *Bazzania calcarata* and *Acromastigum inaequilaterum*, which accounted for 54.3%, 57.8% and 58.3% of total peak areas, respectively. 1-dodecene and 1-tetradecene (**1**) were dominant in *B. asymmetrica*, representing 76.4% of total volatiles.

The headspace of *Cheilolejeunea trifaria* mainly emitted sesquiterpenoids such as cycloheptane 4-methylene-1-methyl-2-(2-methyl-1-propeny-1-yl)-1-vinyl (3.5%), (+)-eremophilene (12%), isoaromadendrene epoxide (2.0%), caryophyllene oxide (4.4%) and (-)-globulol (10%). These compounds accounted for 32% of the total volatiles of *C. trifaria* headspace. β-chamigrene (11.3%) was identified in *B. asymmetrica*. The headspace of *Pycnolejeunea grandiocellata* detected longifolene (7.9%) and cycloheptane 4-methylene-1-methyl-2-(2-methyl-1-propeny-1-yl)-1-vinyl (3.6%). The headspaces of *Pallavicinia lyellii* and *O. pseudorufescens* were dominated by the aromatic hydrocarbons benzothiazole and naphthalene, which represented 65.4% and 75.1%, respectively. The aliphatic aldehydes nonanal and decanal are the commonly identified volatiles in all species. *Pycnolejeunea cavistipula* and *Pallavicinia lyellii* emitted both aldehydes, representing the cumulative area of 15.2% and 9.4%, respectively. Emission of nonanal was detected in *C. trifaria* (2.1%), *Pycnolejeunea grandiocellata* (4.2%) and *B. calcarata* (16.1%), whereas decanal was detected in *D. ternatensis* (5.0%) and *O. pseudorufescens* (14.0%).

## 3. Discussion

Our data on the chemical profiling of VOCs of bryophytes in two different types of forests in Peninsular Malaysia show considerable volatile differences. Alcohol (1-hexanol, 2-ethyl- and 1-dodecanol) is produced in all the highland species from the montane forest of Cameron Highlands and in none of the species from the Lata Belatan lowland forest. Different types of terpenoid, fatty acid and ester compounds are found in the species from both forests. Nevertheless, some similar major compounds are synthesized by species from both forests, such as the volatiles alkane (dodecane, 2,6,11-trimethyldodecane, tetradecane, pentadecane, hexadecane), alkene (1-dodecene, 1-tetradecane), aldehyde (nonanal, decanal) and aromatic hydrocarbon (naphthalene, benzothiazole). All the species of bryophytes from the montane forest have volatile terpenoids (sesquiterpenoids and diterpenes). In contrast, only four species of liverworts from the lowland forest have sesquiterpenoids and none produce diterpenes. The elevational differences in VOC composition correlate with the very different taxonomic composition of the lowland and montane species assemblages in this study. Lowland liverwort species of Lata Belatan are from the families Lepidoziaceae (*Bazzania, Acromastigum*), Lejeuneaceae (*Cheilolejeunea, Drepanolejeunea*, *Pycnolejeunea*), and Pallaviciniaceae (*Pallavicinia*), while the montane species from the Cameron Highlands represent very different families (apart from a few spp. of *Bazzania*), viz. Plagiochilaceae (*Plagiochila*), Lophocoleaceae (*Heteroscyphus*), Frullaniaceae (*Frullania*), Mastigophoraceae (*Mastigophora*) and Scapaniaceae (*Plicanthus*). The elevational pattern detected in the VOC composition of liverworts thus appears to be an evolutionary feature that needs verification at other sites. The distinct differences in VOC composition detected among species at single elevational sites confirm the findings of Ludwiczuk and Asakawa [6], who showed that the secondary metabolite composition of bryophytes is usually species-specific, particularly for sesquiterpenoids in liverworts (see examples in [6]). It has also been shown that the production of VOCs by plants, in general, depends on many factors [7], and that each organism may produce specific VOCs according to their functional role in the ecosystem.

Nine out of 12 species from Cameron Highlands share the same highest component of volatile, i.e., pentanoic acid, 2,2,4-trimethyl-3-carboxyisopropyl, isobutyl ester, with the concentration of the constituents ranging from 16.9–34.6%. *Garovaglia elegans* and *Anthoceros angustus* have the highest concentration, with 32.7% and 34.6%, respectively. Hitherto, the highest number of VOCs in a species of hornworts was found in *Leiosporoceros dussii* (Steph.) Hässel from Panama, which produced two terpenoids and 27 VOCs [8,9]. In contrast, the highest number of VOCs of hornworts detected in the present study was 11 (in *A. angustus*). Interestingly, gametophytes and sporophytes of the hornwort *L. dussii* showed apparent differences in terms of VOCs, with menthacamphor being the main constituent of female thalli (18 VOCs identified in total) while hexanol was the main constituent of male thalli (14 VOCs identified in total) and hexanal in sporophytes (18 VOCs identified in total) [9].

The emission of VOCs by plants is mainly associated with a range of biotic and abiotic stress factors such as high temperature, high light and herbivore attack [10]. Plants produce VOCs for different reasons and are essential to the functioning of ecosystems. Among them are pheromones, eavesdropping and mimicry, plant–insect interaction, plant–plant communication and microorganisms (effect of VOC production in microbial community on plants) [11]. Their function in chemical ecology by a wide array of organisms ranging from animals and microorganisms to fungi has been well-documented and studied in great depth, particularly in insects. However, the chemical ecology of interactions of nonvascular plants such as bryophytes through VOCs has only begun to be explored recently. One of the first studies was in a peatland moss, *Hamatocaulis vernicosus,* and its competitor, *Sphagnum flexuosum,* by Vicherová et al. [7]. They proved that *H. vernicosus* used volatile chemical signal information in neighbor detection. Specifically, *H. vernicosus* can detect the VOCs of *S. flexuosum*, thereby regulating its growth in response to sharing resources such as light and space.

As for plant–insect interactions, VOCs have long been recognized to play a major role in attracting pollinators and offering defense against herbivores. In bryophytes, it has been shown that VOCs may play a role in spore and spermatozoid dispersal. For examples, several species from the moss family Splachnaceae use brightly colored, scented sporophytes to attract flies that facilitate spore dispersal [12]. Similarly, the moss *Ceratodon purpureus* utilizes volatile scents to manipulate microarthropod behavior, increasing moss fertilization [13]. It has also been found that odors of gametophytes and sporophytes of Splachnaceae species differed significantly, with gametophyte odors consisting of sesquiterpenoids and hydrocarbons, whereas sporophyte odors were much more pungent and chemically complex [12]. In some cases, VOCs may serve as chemotaxonomic markers and be used as characters in taxonomy, e.g., in recognition of cryptic species of *Conocephalum conicum* [14]. A summary of the role of VOCs in bryophytes is presented in Table 5.

## 4. Materials and Methods

### 4.1. Plant Material

**Figure 2:**
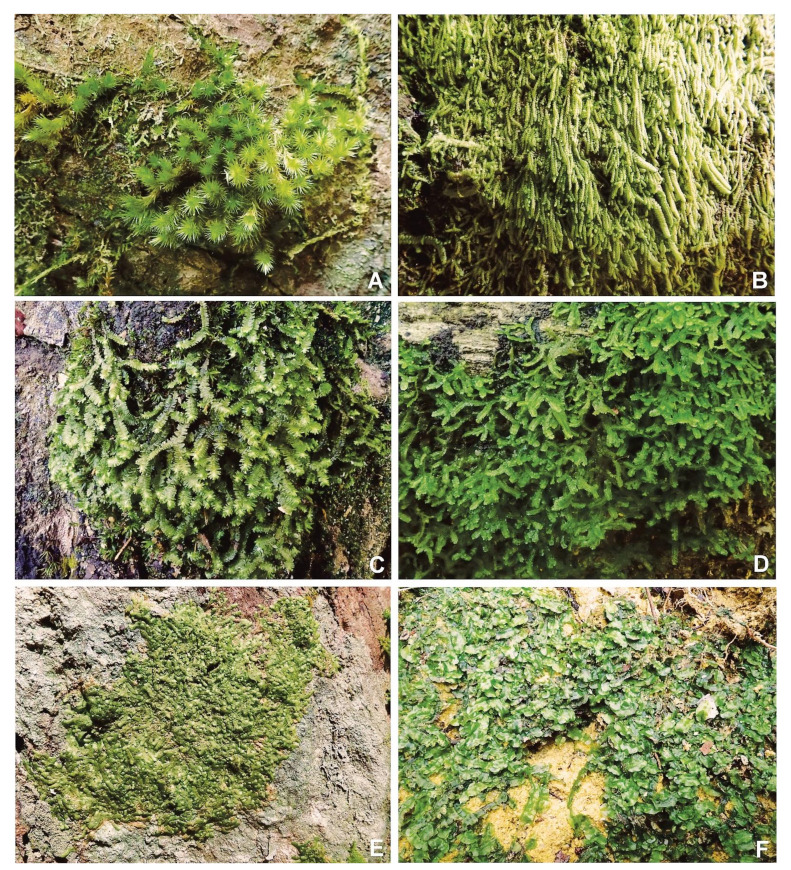
Bryophyte species and their habit from Lata Belatan. (**A**) *Oedicladium pseudorufescens* (Hampe) B.C. Tan and Mohamed. (**B**) *Drepanolejeunea ternatensis* (Gottsche) Schiffn. (**C**) *Bazzania asymmetrica* (Steph.) N. Kitag. (**D**) *Bazzania calcarata* (Sande Lac.) Schiffn. (**E**) *Pycnolejeunea grandiocellata* Steph. (**F**) *Pallavicinia lyellii* (Hook.) Carruth.

**Figure 3:**
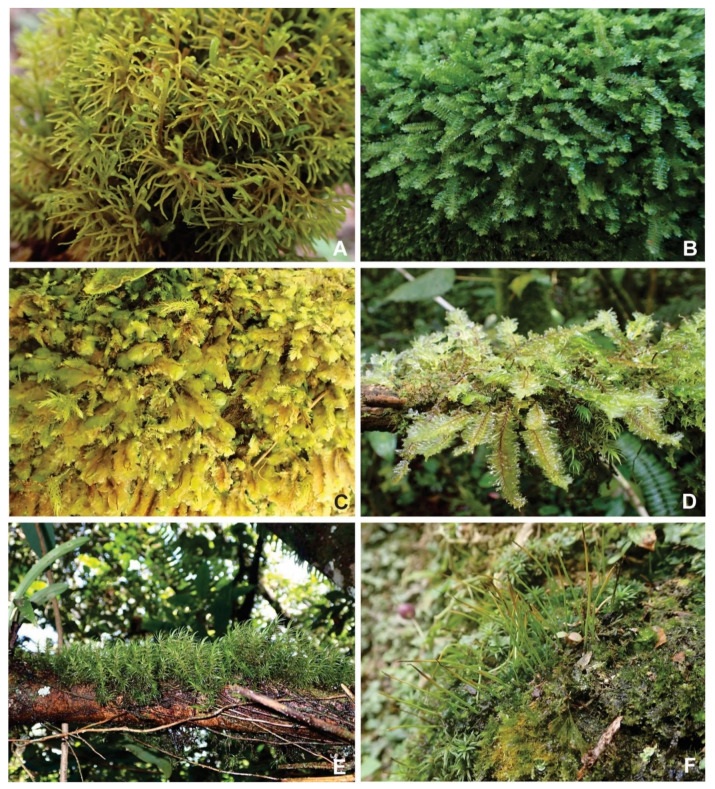
Bryophyte species and their habit from Cameron Highlands. (**A**) *Mastigophora diclados* (F. Weber) Nees. (**B**) *Heteroscyphus coalitus* (Hook.) Schiffn. (**C**) *Distichophyllum mittenii* Bosch and Sande Lac. (**D**) *Plagiochila bantamensis* Dumort. (**E**) *Dicranoloma braunii* (Müll. Hal.) Paris. (**F**) *Anthoceros angustus* Steph.

Plant samples were collected from two study areas, namely Cameron Highlands montane forest, Pahang and Lata Belatan lowland dipterocarp forest, Terengganu (Figure 2 and Figure 3). According to the Malaysian Metrological Department, the temperature of Cameron Highlands is within the range of 13 °C–24 °C throughout the year, and the mean annual rainfall is 2400 mm, while in Lata Belatan, the temperature is 21 °C–32 °C and the yearly rainfall is between 2000–2500 mm. In addition, voucher specimens were identified and deposited in the Herbarium of Universiti Malaysia Terengganu (UMTP). The samples were identified based on morphological characteristics, using identification keys in various publications, e.g. [15,16,17,18,19,20].

### 4.2. Headspace Volatiles Collection

The young green shoots of the bryophyte sample were chosen, excluding the lower part, usually dried up or brown in color. The plant sample was cleaned from the substrate, and other species intermingled together under a stereomicroscope. The sampling of volatiles was carried out using the dynamic headspace technique. Each species was placed on damp cotton in an enclosed glass jar (11 cm × 10 cm). Incoming air was purified by activated charcoal (Sigma-Aldrich, St Louis, MO, USA), and outgoing air was trapped on 150 mg Tenax^®^ TA (60–80 mesh; Sigma-Aldrich) that was connected to a vacuum pump (Rocker430, New Taipei City, Taiwan) at a flow rate of 2 L/min. Volatiles were collected for 24 h. Bryophyte volatiles were extracted by eluting the Tenax^®^ TA with 1 mL hexane, containing 5 pg/µL of benzyl acetate (Sigma-Aldrich) as internal standard. For control, volatiles were also collected from a glass jar containing damp cotton.

### 4.3. Gas Chromatography–Mass Spectrometry (GCMS)

The volatiles of bryophytes were analyzed using SHIMADZU QP2010 Ultra gas chromatograph–mass spectrometer. A splitless injection of 1 µL was carried out with the GC injector set to 300 °C. Compounds were separated on a Zebron ZB-5ms column (30 m × 250 µm i.d. × 0.25 µm film thickness; Phenomenex). The GC oven was programmed as follows: the initial temperature was kept at 50 °C for 1 min and heated at a rate of 5 °C min^−1^ to 300 °C. Then, the temperature was increased to 320 °C at 5 ℃ min^−1^ and was maintained for 5 min. Helium was used as the carrier gas at a flow rate of 1 mL/min. Injection temperature was set at 300 °C, and injection volume of 1 µL in splitless mode. The temperature of the ion source was set at 200 °C. All data were collected from full scan mass spectra in 50 to 600 *m*/*z* at 70 ev.

### 4.4. Data Analysis

Identification of compounds was carried out by comparison of mass spectra with NIST library spectra. However, a single extract containing complex VOCs cannot be distinguished by mass spectra alone. Together with mass spectrometry, retention indices (RI) provide nearly precise identification of isomers. The RI was calculated using the following equation [21]:

RI = 100n + 100(t_x_ − t_n_)/(t_n+1_ − t_n_)

where RI is the retention index for temperature-programmed gas chromatography; t_n_ and t_n_ are the retention times of the n-alkane eluting immediately before and after target compound, x, respectively; t_x_ is the retention time of compound x; and n is the number of carbon atoms of the n-alkane eluting before the target compound. The n-alkane standard, C_7_-C_30_ (Sigma-Aldrich), was used and run using the parameters described above prior to GCMS analysis. The experimental RI was compared with reported RI in literature [22], Pherobase (https://www.pherobase.com, accessed on 11 April 2022) and NIST (https://webbook.nist.gov/chemistry/, accessed on 11 April 2022). The volatile composition was expressed in the percentage of peak area relative to the total peak area of each compound.

## 5. Conclusions

Our study provides VOC profiling of selected Peninsular Malaysian bryophytes in two different types of forests: montane forest and lowland dipterocarp forest. The results show apparent volatile differences in their composition, indicating that the secondary metabolites of bryophytes are usually species-specific and could be an evolutionary feature at the family level. However, VOCs and their function and development in bryophytes are poorly understood, unlike other vascular plant groups. Hence, future research on the volatile composition of bryophytes, particularly from the liverwort oil bodies that accumulate and contain bioactive compounds, may shed some light on their existence, emission and function of the VOCs in response to biotic and abiotic stresses.

## Figures and Tables

**Table 1:** Bryophyte species from Cameron Highlands with information on the family and species name, collecting locality and substrate.

Family	Species	Locality	Substrate
Anthocerotaceae	*Anthoceros angustus* Steph.	Near Arcadia Cottage, along the forest trail to the entrance of Jungle Walk Trail 3 to Mount Berembun, at 1482 m alt., N4°29.106′, E101°23.005′	Soil
Dicranaceae	*Dicranoloma braunii* (Müll. Hal.) Paris	Along the forest trail at Sungai Pauh Campsite to Mount Berembun, a lower montane forest, rich in bryophytes, at 1470–1510 m alt., N4°28.797′, E101°23.162′	Tree trunk
Fissidentaceae	*Fissidens crispulus* Brid.	Along the forest trail to Parit Falls, a lower montane rainforest at streamside, at 1430–1470 m alt., N4°28.476′, E101°23.026′	Soil
Hookeriaceae	*Distichophyllum mittenii* Bosch and Sande Lac.	Along the forest trail to Parit Falls, a lower montane rainforest at streamside, at 1430–1470 m alt., N4°28.476′, E101°23.026′	Tree trunk
Pterobryaceae	*Garovaglia elegans* (Dozy and Molk.) Bosch and Sande Lac.	Along the forest trail at Sungai Pauh Campsite to Mount Berembun, a lower montane forest, rich in bryophytes, at 1470–1510 m alt., N4°28.797′, E101°23.162′	Tree trunk
Frullaniaceae	*Frullania apiculata* (Reinw., Blume and Nees) Nees	Along the forest trail to Parit Falls, a lower montane rainforest at streamside, at 1430–1470 m alt., N4°28.476′, E101°23.026′	Tree trunk
Lepidoziaceae	*Bazzania longicaulis* (Sande Lac.) Schiffn.	Along the forest trail to Parit Falls, a lower montane rainforest at streamside, at 1430–1470 m alt., N4°28.476′, E101°23.026′	Soil
	*Bazzania loricata* (Reinw., Blume and Nees) Trevis.	Along the forest trail at Sungai Pauh Campsite to Mount Berembun, a lower montane forest, rich in bryophytes, at 1470–1510 m alt., N4°28.797′, E101°23.162′	Soil
Lophocoleaceae	*Heteroscyphus coalitus* (Hook.) Schiffn.	Along the forest trail to Parit Falls, a lower montane rainforest at streamside, at 1430–1470 m alt., N4°28.476′, E101°23.026′	Rotten log
Mastigophoraceae	*Mastigophora diclados* (F. Weber) Nees	Along the forest trail at Sungai Pauh Campsite to Mount Berembun, a lower montane forest, rich in bryophytes, at 1470–1510 m alt., N4°28.797′, E101°23.162′	Soil
Plagiochilaceae	*Plagiochila bantamensis* Dumort.	Along the forest trail at Sungai Pauh Campsite to Mount Berembun, a lower montane forest, rich in bryophytes, at 1470–1510 m alt., N4°28.797′, E101°23.162′	Tree branch
Scapaniaceae	*Plicanthus hirtellus* (F. Weber) R.M. Schust.	Along the forest trail at Sungai Pauh Campsite to Mount Berembun, a lower montane forest, rich in bryophytes, at 1470–1510 m alt., N4°28.797′, E101°23.162′	Fallen log

**Table 2:** Major volatile compounds identified in bryophyte headspace from Cameron Highlands. RT: retention time; RI: retention index; RI_ref_: retention index in reference. Mosses—M1: *Dicranoloma braunii*, M2: *Fissidens crispulus*, M3: *Distichophyllum mittenii*, M4: *Garovaglia elegans*. Liverworts—L1: *Heteroscyphus coalitus*, L2: *Frullania apiculata*, L3: *Plicanthus hirtellus*, L4: *Bazzania longicaulis*, L5: *Plagiochila bantamensis*, L6: *Bazzania loricata*, L7: *Mastigophora diclados*. Hornwort—H1: *Anthoceros angustus*.

No.	Compound	RT	RI	RI_ref_	Peak Area (%)											
					Moss				Liverwort							Hornwort
					M1	M2	M3	M4	L1	L2	L3	L4	L5	L6	L7	H1
	**Alkane**															
1	Dodecane	14.48	1201	1200		3.5		2.4		3.4	3.4	14.1		2.9	2.3	3.6
2	2,6,11-trimethyldodecane	16.55	1276	1275								5.5				
3	Tetradecane	19.96	1400	1400			4.5	3.0	3.8	3.7		7.8		3.4	3.8	4.2
4	Pentadecane	22.49	1500	1500											1.5	
5	Hexadecane	24.90	1600	1600					2.4		3.3			2.5	2.1	
	**Alkene**															
6	6-Butyl-1,4-cycloheptadiene	13.66	1174	1165									8.9	1.7		
7	1-dodecene	14.24	1193	1192	12.1	7.4	8.4	3.1	6.9	6.9	3.2			2.6	3.5	
8	1-tetradecene	19.75	1393	1389	18.2	11.5	11.7	5.8	11.7	11.6	6.3		12.2	4.5	6.2	6.7
	**Alcohol**															
9	1-Hexanol, 2-ethyl-	9.50	1032	1031	41.2	7.2	4.7		8.2	8.0	4.4	26.0	6.7	3.1	2.6	2.8
10	1-Dodecanol	21.86	1476	1473				3.1				5.3	9.2	2.7	2.7	
	**Aldehyde**															
11	Nonanal	11.73	1107	1104	7.6			9.7			8.3			8.3	8.5	10.4
12	Decanal	14.68	1208	1205		7.7	8.5	10.5	7.7	6.8	10.7	7.5		9.4	9.1	12.2
13	2-methyl-3-phenyl-propanal	15.78	1249	1245											2.3	
	**Aromatic hydrocarbon**															
14	Naphthalene	14.14	1190	1186	9.3	13.2	8.5	4.2	3.9	5.7	5.1	4.1		7.7		8.2
15	Benzothiazole	15.31	1232	1230	6.7				6.3	3.8	4.1					
16	Anethole	17.0	1291	1289										2.5		
	**Fatty acid and Ester**															
17	Methyl salicylate	14.31	1195	1193		3.2				3.1	2.3			2.6	2.3	
18	Pentanoic acid, 2,2,4-trimethyl-3-carboxyisopropyl, isobutyl ester	24.61	1589	1588		16.9	21.8	32.7	18.4	22.7	27.9	16.7	4.5	27.5	25.2	34.6
19	1,1’-biphenyl, 2,2’,5,5’-tetramethyl-	26.67	1679	1669				4.0			4.1			3.6	3.0	4.7
20	Isopropyl palmitate	33.82	2024	2024										1.9		
	**Terpenoid**															
21	Limonene	9.60	1035	1032		10.3	17.8	6.6	12.4	13.3	9.0	12.9	17.4	3.3	9.1	6.3
22	Linalool	11.58	1101	1099					6.5	5.6				2.7	4.4	
23	Menthol	13.90	1182	1176	4.8		8.8		6.9	5.4						
24	β-elemene	18.21	1337	1375				6.1					17.7			
25	1,8-dimethyl-4-(prop-1-en-2-yl)spiro[4.5]dec-7-ene	21.98	1481	1474									6.5			
26	Germacrene B	22.48	1500	1502									10.4			
27	2,6,10- trimethyl pentadecane	25.97	1648	1647											2.1	
28	Kaur-16-ene	34.46	2058	2059										2.4		
	**Other compounds**															
29	Geranylacetone	21.18	1450	1451				5.3			3.3			4.5	3.6	4.4
30	Tetradecamethyl- cycloheptasiloxane	21.56	1465	1468		15.7									5.6	

**Table 3:** Bryophyte species from Lata Belatan with information on the family and species name, collecting locality and substrate.

Family	Species	Locality	Substrate
Myuriaceae	*Oedicladium pseudorufescens* (Hampe) B.C. Tan and Mohamed	Along the forest trail at the Lata Belatan Recreational Forest and waterfall, situated at the base of Mount Tebu, at 100–200 m alt., N5°37.900’, E102°35.753’	Tree branch
Lejeuneaceae	*Cheilolejeunea trifaria* (Reinw., Blume and Nees) Mizut.	Along the forest trail at the Lata Belatan Recreational Forest and waterfall, situated at the base of Mount Tebu, at 100–200 m alt., N5°37.900’, E102°35.753’	Tree trunk
	*Drepanolejeunea ternatensis* (Gottsche) Schiffn.	Along the forest trail at the Lata Belatan Recreational Forest and waterfall, situated at the base of Mount Tebu, at 100–200 m alt., N5°37.900’, E102°35.753’	Tree trunk
	*Pycnolejeunea cavistipula* (Steph.) Mizut.	Along the forest trail at the Lata Belatan Recreational Forest and waterfall, situated at the base of Mount Tebu, at 100–200 m alt., N5°37.900’, E102°35.753’	Tree trunk
	*Pycnolejeunea grandiocellata* Steph.	Along the forest trail at the Lata Belatan Recreational Forest and waterfall, situated at the base of Mount Tebu, at 100–200 m alt., N5°37.900’, E102°35.753’	Tree trunk
Lepidoziaceae	*Acromastigum inaequilaterum* (Lehm. and Lindenb.) A. Evans	Along the forest trail at the Lata Belatan Recreational Forest and waterfall, situated at the base of Mount Tebu, at 100–200 m alt., N5°37.900’, E102°35.753’	Tree root
	*Bazzania asymmetrica* (Steph.) N. Kitag.	Along the forest trail at the Lata Belatan Recreational Forest and waterfall, situated at the base of Mount Tebu, at 100–200 m alt., N5°37.900’, E102°35.753’	Tree trunk
	*Bazzania calcarata* (Sande Lac.) Schiffn.	Along the forest trail at the Lata Belatan Recreational Forest and waterfall, situated at the base of Mount Tebu, at 100–200 m alt., N5°37.900’, E102°35.753’	Tree trunk
Pallaviciniaceae	*Pallavicinia lyellii* (Hook.) Carruth.	Along the forest trail at the Lata Belatan Recreational Forest and waterfall, situated at the base of Mount Tebu, at 100–200 m alt., N5°37.900’, E102°35.753’	Soil

**Table 4:** Major volatile compounds identified in bryophyte headspace from Lata Belatan. RT: retention time; RI: retention index; RI_ref_: retention index in reference. Mosses—M1: *Oedicladium pseudorufescens*. Liverworts—L1: *Drepanolejeunea ternatensis*, L2: *Pallavicinia lyellii*, L3: *Bazzania asymmetrica*, L4: *Pycnolejeunea cavistipula*, L5: *Cheilolejeunea trifaria*, L6: *Pycnolejeunea grandiocellata,* L7: *Bazzania calcarata*, L8: *Acromastigum inaequilaterum*.

No.	Compound	RT	RI	RI_ref_	Peak Area (%)								
					Moss	Liverwort							
					M1	L1	L2	L3	L4	L5	L6	L7	L8
	Alkane												
1	2,4-dimethylheptane	4.32	823	822								14.1	
2	Decane	8.70	1001	1000								4.6	
3	4,5-dimethylnonane	10.34	1062	1035								7.7	
4	Dodecane	14.48	1200	1200								13.1	
5	2,6,11-trimethyldodecane	16.55	1322	1275									37.0
6	Tridecane	17.28	1300	1300			2.4						
7	Tetradecane	19.96	1400	1400								12.4	21.3
8	Pentadecane	22.49	1500	1500			5.0				51.5		
9	Hexadecane	24.90	1600	1600							2.8	5.9	
	**Alkene**												
10	1-dodecene	14.24	1193	1192		8.0	4.3	32.2			18.6		
11	1-tetradecene	19.75	1393	1389	6.0	12.9	10.6	44.2	11.8				40.7
	**Aldehyde**												
12	Nonanal	11.73	1106	1104			5.3		9.9	2.1	4.2	16.1	
13	Decanal	14.68	1208	1205	14.0		4.1		6.3				
14	Dodecanal	20.23	1412	1413	1.7	8.0							
15	Pentadecanal	27.53	1717	1711	3.1								
	**Aromatic hydrocarbon**												
16	Naphthalene	14.14	1190	1186	67.9	64.2	62.0		68.9	3.4	7.8		
17	Benzothiazole	15.31	1232	1230	7.2		3.4					6.8	
	**Fatty acid and esters**												
18	Cyclopentanecarboxylic acid, 3-isopropylidene-, bornyl ester	26.83	1685	-						24.5			
19	2-ethylhexyl salicylate	29.44	1805	1805								3.8	
	**Terpenoid**												
20	Cycloheptane 4-methylene-1-methyl-2-(2-methyl-1-propen-1-yl)-1-vinyl-	20.35	1417	-						3.5	3.6		
21	Longifolene-(V4)	20.58	1426	1427							7.9		
22	β-chamigrene	22.16	1486	1479				11.3					
23	(+)-eremophilene	22.47	1499	1498						12.0			
24	Isoaromadendrene epoxide	24.24	1574	1579						2.0			
25	Caryophyllene oxide	24.59	1588	1581						4.4			
26	(-)-globulol	25.10	1610	1604						10.0			
	**Other compounds**												
27	1-(1-Ethyl-2,3-dimethyl-cyclopent-2-enyl)-ethanone	22.00	1482	-						29.9			
28	Cyclopentane, 1,1,3-trimethyl-3-(2-methyl-2-propenyl)-	23.78	1555	-				12.2		6.1			
29	Benzophenone	25.66	1635	1636						2.0	3.7	6.5	
30	1,2,3,6-tetramethylbicyclo [2.2.2] octane	26.11	1653	-								9.0	

**Table 5:** A summary of the role of volatile organic compounds (VOCs) in bryophytes.

Bryophytes	Role of VOCs	References
Splachnaceae	Manipulation of insect behavior to facilitate spore dispersal	Marino et al. [12]
*Ceratodon purpureus* (Hedw.) Brid.	Manipulation of microarthropods to increase spermatozoid dispersal	Rosenstiel et al. [13]
*Conocephalum conicum* (L.) Dumort.	Taxonomic characterization of cryptic species	Ludwiczuk et al. [14]
*Hamatocaulis vernicosus* (Mitt.) Hedenäs and *Sphagnum flexuosum* Dozy and Molk.	Plant neighbor detection	Vicherová et al. [7]

## Data Availability

No additional data are available.
